# A Four-Year Study of Antibiotic Resistance, Prevalence and Biofilm-Forming Ability of Uropathogens Isolated from Community- and Hospital-Acquired Urinary Tract Infections in Southern Italy

**DOI:** 10.3390/pathogens14010059

**Published:** 2025-01-11

**Authors:** Marilena Trinchera, Angelina Midiri, Giuseppe Mancuso, Maria Antonietta Lagrotteria, Carmelo Antonio De Ani, Carmelo Biondo

**Affiliations:** Department of Human Pathology, University of Messina, 98125 Messina, Italy; tmarilena08@gmail.com (M.T.); amidiri@unime.it (A.M.); mancusog@unime.it (G.M.); antolagrotteria@hotmail.com (M.A.L.); carmelo.deani@studenti.unime.it (C.A.D.A.)

**Keywords:** hospital-acquired urinary tract infections (HA-UTI), community-acquired urinary tract infections (CA-UTI), biofilm, MDR

## Abstract

The aim of this study was to investigate the differences between nosocomial and community microorganisms isolated from patients with UTI by determining their bacterial profile, antibiotic resistance and ability to produce biofilms. A retrospective study, based on bacterial isolates from consecutive urine samples collected between January 2019 and December 2023, was conducted at a university hospital. The main pathogens isolated from both community and hospital samples were the same, but their frequency of isolation differed. Compared with community-associated cases, hospital-associated infections have more isolates of *Acinetobacter baumanii complex*. In contrast, *Proteus mirabilis* isolates were more prevalent in community than in hospital infections. In both hospital and community isolates, gram-positive bacteria showed a lower overall antimicrobial resistance (22%) compared to gram-negative bacteria (30%). The data demonstrated that individual strains exhibited disparate degrees of capacity for biofilm formation. Additionally, the data indicate an inverse correlation between biofilm production and antibiotic resistance. Isolates from community patients exhibited lower capacities for biofilm production in comparison to the capacities demonstrated by microorganisms isolated from nosocomial patients (29% and 35%, respectively). Area-specific surveillance studies can provide valuable information on UTI pathogens and antimicrobial resistance patterns, which can be useful in guiding empirical treatment.

## 1. Introduction

Urinary tract infections (UTIs) are prevalent bacterial infections that affect millions of people worldwide each year [1]. They occur in both community and healthcare settings and result in significant morbidity and high medical costs. In the United States, recurrent UTIs have been estimated to cost over 5 billion US dollars annually [2]. About a third of women have experienced at least one symptomatic urinary tract infection (UTI), while 20–30% of women experience recurrent UTIs (that is, two or more infections within six months or three or more infections within one year) [3]. Older men and women are at increased risk of infection due to predisposing conditions such as obstructive prostate uropathy in men and poor bladder emptying due to uterine prolapse in women [4]. In general, UTIs are classified based on the location of the infection. These locations include the urethra (urethritis), ureters (ureteritis), bladder (cystitis) and kidneys (pyelonephritis) [5]. Urinary tract infections are further categorized by whether there is a predisposition to infection (uncomplicated or complicated) or whether they are primary or recurrent. Most uncomplicated UTIs are caused by uropathogens that reside in the bowel and migrate to colonize the bladder after occasional urethral contamination [6]. A complicated UTI is defined as one where there is an abnormality of the urinary tract that makes it more susceptible to infection, such as a catheter, neurogenic bladder, renal insufficiency, or the presence of stones [6]. In the United States, 70–80% of complicated urinary tract infections (UTIs) are associated with the use of catheters—an association that leads to longer hospital stays and more extensive treatment [7]. Bacterial uropathogens such as *Escherichia coli*, *Klebsiella pneumoniae*, *P. mirabilis*, *Enterococcus faecalis* and *Staphylococcus* spp. are the main causes of UTIs [8]. Although less common, some fungal species can also cause UTIs [9]. UTIs are a group of clinical disorders with varying clinical presentations. The range of UTIs includes asymptomatic bacteriuria, which is the presence of bacteria in the urine without any specific UTI symptoms and does not require treatment except in particular cases (e.g., pregnant women, neutropenic patients), and symptomatic UTIs that require antibiotic treatment [10]. Thus, asymptomatic bacteriuria does not meet the diagnostic criteria for a urinary tract infection. In addition, the lack of leukocyturia is crucial in differentiating asymptomatic bacteriuria from conventional UTIs. Antibiotics are often chosen empirically to initiate prompt therapy, as the causative agent and relative antibiotic sensitivity cannot be identified before 48–72 h, although it is possible to achieve this earlier, depending on the lab technique used [11]. However, this approach can lead to the selection of increasingly resistant bacteria if the wrong choice is made [10]. Mismanagement in drug use, often self-prescribed, low-dose and for long periods, can exacerbate this condition, especially in community infections [12]. Over the past two decades, there has been a significant increase in UTIs caused by antibiotic-resistant gram-negative bacteria [13,14]. This is due to the limited treatment options resulting from the development of multidrug resistance. (MDR) [15]. Between 2016 and 2019, the European Society for Disease Prevention and Control (ECDC) reported an increase in the number of cases of antibiotic-resistant bacterial infections, which resulted in over 35,000 deaths in Europe [15]. According to 2015 data from the ECDC, Italy has the highest incidence of infections caused by multi-drug-resistant microorganisms in Europe, both in terms of cases and deaths [16]. The etiological agents responsible for UTIs and their resistance profiles vary across different geographical regions [17]. Furthermore, it has been observed that, alongside known resistance mechanisms such as decreased membrane permeability, mutations of target molecules, overexpression of efflux pumps and inactivation of antibiotic molecules, approximately 60–80% of the bacteria responsible for UTIs are capable of producing a biofilm, a polysaccharide matrix which, at an extracellular level, mediates adhesion to the uroepithelium and/or indwelling devices (catheters), allowing for better colonization of surfaces and evasion of the immune system [17,18]. But this extracellular matrix also has the property of increasing microbial resistance to antibiotics by acting as a poorly penetrable barrier and creating a microenvironment that facilitates communications between bacteria through biochemical signals, including the horizontal transmission of resistance genes [19]. However, resistance to antibiotics within the biofilm can also be determined by the cooperative abilities of individual cells capable of changing their phenotypic characteristics [20]. In fact, the sessile forms grow much more slowly than planktonic cells, thus slowing down the intake of antibiotics [21]. It can be deduced that the production of biofilms further complicates the current situation regarding antibiotic resistance, which affects the entire public health system, both in terms of morbidity and economics. Therefore, it is crucial to have a comprehensive understanding of the prevalence of infectious agents and the relative presence of resistance mechanisms in both hospital and community settings at the local level, as well as the isolation and identification of the relevant biofilm-producing microorganisms to avoid disadvantageous therapeutic efforts, as they could exacerbate the condition of multidrug resistance [22]. The identification of the biofilm phenomenon may facilitate more efficacious control and prevention of CAUTI infections [23]. The implementation of a systematic monitoring program would be advantageous, given that the etiologic agents and resistance patterns can change over time. The aim of this study was twofold: to examine the differences between nosocomial and community-acquired microorganisms isolated from patients with UTI and to identify the presence of biofilm production in uropathogenic isolates by determining the resistance profiles associated with its formation. Moreover, our study provides data on the prevalence and antimicrobial susceptibility patterns within our geographic region, with the objective of formulating guidelines for the appropriate utilization of antibiotics.

## 2. Materials and Methods

### 2.1. Urine Specimen Collection

A retrospective study was conducted on bacterial isolates from urine samples collected at a university hospital in southern Italy between January 2019 and December 2023. This study included all patients who presented with clinical symptoms. Infections that developed 48 h after admission were classified as nosocomial infections, according to the definition provided by the CDC [24]. The absence of hospitalization in the last 15 days was used to determine whether a patient’s infection was acquired in the community or in the hospital. Informed consent for participation was obtained from all subject. Urine samples were inoculated onto cystine lactose electrolyte deficient agar (CLED) and incubated for 24 h at 37 °C according to the manufacturer’s instructions [25]. The urine specimens were classified into four categories: positive, contaminated, nonsignificant and no growth. Positive specimens had significant growth of one or two uropathogens, with a concentration of at least 10^5^ colony-forming units per milliliter of urine. Contaminated specimens were characterized by the presence of three or more bacterial species in concentrations above the threshold. Nonsignificant specimens had bacterial species growing in concentrations below the threshold. Specimens classified as ‘no growth’, resulting in a negative urine culture, had no growth detected.

### 2.2. Bacterial Identification and Antibiotic Test

Identification and antibiotic susceptibility testing were performed using the Vitek-2 System with VITEK^®^2 (BioMérieux, Paris, France) and GN and GP cards, as previously described [21]. The antimicrobial susceptibility profile of the isolates was confirmed using VITEK tests as previously described, and the same tests were used to screen for ESBL producers and to detect carbapenemase production [26]. The data were interpreted according to the clinical breakpoints outlined in the EUCAST 2020 criteria [27]. Duplicate data were reviewed and eliminated according to guidelines from the Clinical and Laboratory Standards Institute (CLSI, 2014) and the European Antimicrobial Resistance Surveillance Network (ECDC, 2013). This included isolates from the same patient, specimen, ward, or species, or those with similar antibiotic patterns within 15 days. The following antibiotics were selected from the literature: cefotaxime (third-generation cephalosporins), fosfomycin (epoxides), trimethoprim-sulfamethoxazole (sulfonamides), nitrofurantoin (nitrofurans), amoxicillin/clavulanate (aminopenicillin/BLI), imipenem (carbapenems), ciprofloxacin (quinolones) and gentamicin (aminoglycosides) [28,29,30]. As previously reported, MDR, which stands for ‘multidrug resistance’, indicates resistance to at least one agent from three or more antimicrobial categories [14].

### 2.3. Biofilm Formation in Bacterial Isolates from Urine Samples

Two distinct methodologies were employed to ascertain the capacity of the isolates to form biofilms. Two phenotypic techniques were utilized for the purpose of biofilm formation evaluation: the quantitative microtiter plate method and the qualitative evaluation method. The first of these was the 96-well microtiter plate method, and the second was the tube test. The former approach was first described by Stepanović et al. (2007), whereas the latter was initially defined by Christensen et al. (1995) [31,32]. The capacity of the isolates to form biofilms was evaluated using the crystal violet staining method in a 96-well microtiter plate. Briefly, 100 µL of each culture at a concentration of 10^6^ CFU mL^−1^ was pipetted into the wells of a 96-well polystyrene microplate. After incubation for 24 h at 37 °C, the microplates were fixed in absolute methanol for 15 min and air-dried before 200 µL of 1% crystal violet (CV) solution (Sigma-Aldrich, St. Louis, MO, USA) was added to each well. After 15 min of incubation at room temperature, 300 µL of acetic acid (30% *v*/*v*) was added to each well. Plates were then read at 570 nm using a plate reader (DAS, Rome, Italy). Between each step, the wells were washed three times with PBS. The quantification of biofilms in microplates was performed in accordance with the previously established methodology [26]. For the test tube, the samples were plated in Tryptic Soy Agar (TSA) and left to incubate overnight at 37 °C. The colonies were used to prepare a suspension of 3 OD McFarland (3 mL of saline), of which 100 μL was inoculated into a tube with 2.9 mL of Tryptic Soy Broth (TSB) and incubated at 37 °C for 24 h. The tubes, whose broth showed evident turbidity, were decanted and washed with PBS (or saline) and left to dry upside down, and 1 mL of methanol was added to the tubes to fix the sample for 15 min and then removed. At this point, the dried test tubes were stained with 1 mL of crystal violet (1%) for 15 min. The excess color was eliminated by washing with deionized water, and the tubes were left to dry again. The formation of the biofilm was determined by the addition of 1 mL of acetic acid (33% *v*/*v*) and subsequent reading of the respective OD. The tubes were analyzed and rated for strong, moderate and no biofilm formation. One tube containing only culture broth (TSB) was run as a negative control. Experiments were performed in triplicate.

### 2.4. Data Analysis

Odds ratios were used to test the differences in antibiotic resistance between groups and the strength of the association. Only results with *p*-values of less than 0.05 were considered significant. The study used the odds ratio (OR) to assess whether hospitalization was a risk factor for higher antibiotic resistance. An OR greater than 1 indicated a positive association between hospitalization and the outcome, while an OR less than 1 indicated a negative association [33].

## 3. Results

### 3.1. Hospital and Community Uropathogen Isolates

**Figure 1:**
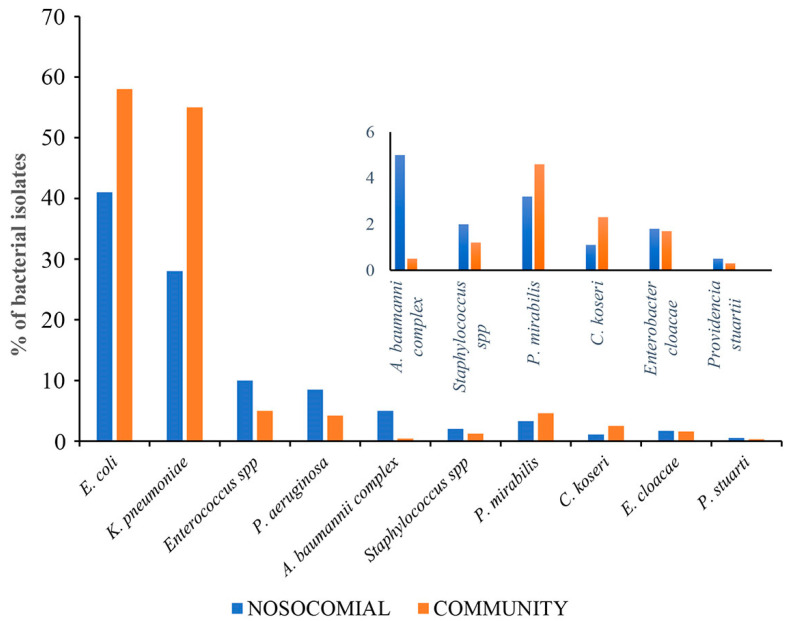
Differences between major uropathogens isolated from nosocomial and community samples.

This study analyzed 15,800 urine samples over a 4-year period from 2019 to 2023. Of these, 4145 (26%) were from the community (CA-UTI), and 11,655 (74%) were from nosocomial samples (H-UTI). The study included patients of both sexes. Of the samples tested, growth of ≥10^5^ colony-forming units per milliliter after overnight incubation was observed in 3547 (27%) CA-UTI and 9528 (73%) H-UTI samples. Of the positive samples, 3009 were tested for the major microbes, of which 2035 (68%) were categorized as nosocomial and 974 (32%) as community. At the hospital level, the samples were evenly distributed between male and female patients. A higher percentage of positive samples was found in female patients (13.2%) compared to male patients (10.2%) in the 0–12 age group. In the 13–50 age group, 103 (13.2%) samples were from female patients, compared to 61 (8%) from male patients. It is worth noting that UTI infections were more prevalent in the over-50 age group, with 649 (82%) samples from male patients and 575 (74%) samples from female patients. At the community level, UTI was more common in females, with 424 (59%) samples, compared to males, with 298 (41%) samples. The age group with the lowest representation was 0–12 years, with 9 (2%) female and 12 (4%) male samples. In the 13–50 age group, the difference between females and males was even more pronounced, with 116 (27.3%) and 29 (10%) positive samples, respectively. In contrast to the data on nosocomial origin, the majority of UTI infections in the over-50 age group were found in females with 299 (41%) samples compared to males with 257 (36%) samples. The most common pathogens isolated from positive samples in both nosocomial and community settings were gram-negative. In H-UTI samples, 1868 (90%) were gram-negative versus 215 (9%) gram-positive. In CA-UTI samples, 922 (95%) were gram-negative, while only 53 (5%) were gram-positive. The most common bacteria isolated from the community samples were *E. coli* 567 (58%), *K. pneumoniae* 234 (24%), *Enterococcus* spp. 44 (5%), *P. mirabilis* 37 (4%), *Pseudomonas aeruginosa* 39 (4%), *Citrobacter koseri* 23 (2.4%), *Enterobacter cloacae* 14 (1.4%), *Staphylococcus* spp. 12 (1.2%), *A. baumannii complex* 3 (0.30%) and *Providencia stuartii* 2 (0.20%). The distribution of species found in nosocomial samples was similar, but their isolation frequency differed. In the hospital, *Enterococcus* spp. (10%), *P. aeruginosa* (8.5%), *E. cloacae* (1.7%) and *P. stuartii* (0.51%) were more frequently isolated than in the community. On the other hand, *E. coli* (39%), *K. pneumoniae* (29.9%), *P. mirabilis* (3.3%) and *C. koseri* (1.1%) were less frequent in the hospital than in the community. A higher prevalence of *A. baumannii complex* (5%) and *Staphylococcus* spp. (2%) isolates was observed in nosocomial infections compared with community-associated cases (Figure 1). Over the four-year period, *E. coli* was the most commonly isolated pathogen, followed by *K. pneumoniae*, *E. faecalis*, *P. aeruginosa* and *E. faecium*, as shown in Table 1. Among hospital inpatients, a significant decrease in the frequency of *E. coli* was observed over time, with isolation rates ranging from 14% to 9% (*p* < 0.0001 in 2019–2023). As shown in Table 1 (upper panel), there was a significant decrease in the frequency of isolation for *E. faecium* (from 1.03% in 2019 to 0.2% in 2023, with *p* = 0.011) and *E. faecalis* (from 1.5% in 2019 to 1.2% in 2023, with *p* = 0.044). No significant changes were observed in *P. aeruginosa*. The most common pathogens isolated from community outpatients over the four-year period are shown in Table 1 (lower panel). The most frequently isolated organisms in this patient group were *E. coli*, followed by *K. pneumoniae* and *E. faecalis*. Notably, the isolation rate of *E. coli* decreased significantly from 2019 to 2020 (*p* < 0.0001) and from 2019 to 2022 (*p* = 0.003). The isolation rate of *P. aeruginosa* decreased from 1.1% to 0.9% between 2019 and 2023. Throughout the study period, the isolation rate of *K. pneumoniae* increased from 4.1% to 7.5% (*p* = 0.325). However, it is important to note that this increase was not statistically significant. Significant changes were not observed in the isolation of *E. faecalis*, while *E. faecium* was not detected in the period between 2020 and 2023 (Table 1 lower panel).

### 3.2. Antimicrobial Susceptibility Patterns from 2019 to 2023

**Figure 2:**
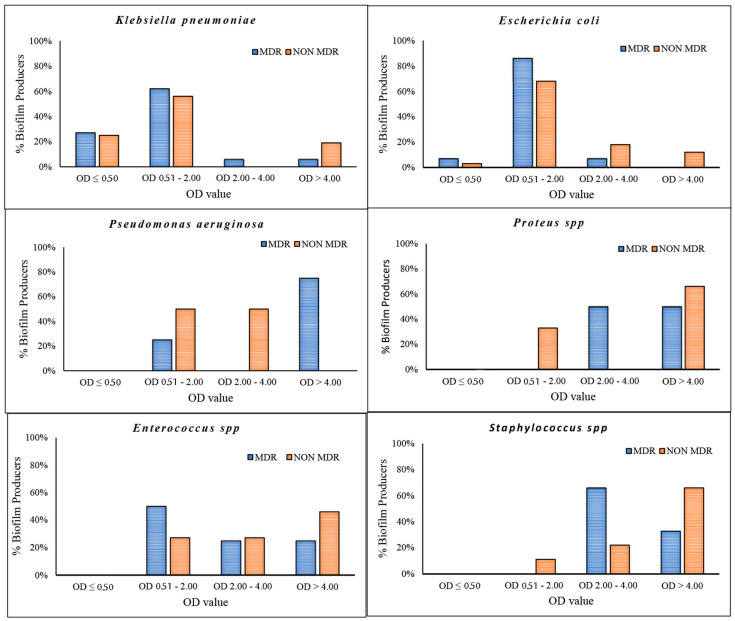
Percentage of biofilm formation detected in *K. pneumoniae*, *E. coli*, *P. aeruginosa*, *Proteus* spp., *Enterococcus* spp. and *Staphylococcus* spp. Isolates were divided into MDR and NON-MDR. OD < 50 = non-biofilm producers; OD 0.50–2.00 = low biofilm producers; OD 2.00–4.00 = medium biofilm producers; OD > 4.00 = strong biofilm producers.

Differences in resistance rates between community- and hospital-acquired uropathogens are shown in Table 2. In both hospital and community isolates, gram-negative bacteria had higher overall antimicrobial resistance than gram-positive bacteria. The differences in resistance rates between gram-negative isolates from outpatient and inpatient UTI samples are shown in Table 2, panels A and B. The most active antimicrobial agents against most uropathogens isolated both in the community and in the hospital were imipenem (C 23%–H 19%) and aminoglycosides (C 7.3%–H 19%) (Table 3 panel A). *P. aeruginosa* and *E. coli* isolates from hospital patients showed no significant differences in resistance compared to those isolated in the community (Table 3 panel B). In hospitalized patients, *K. pneumoniae* was 1.8 times more resistant to trimethoprim-sulfamethoxazole (OR 1.84, *p* = 0.0036), ciprofloxacin (OR 1.93, *p* = 0.0008), imipenem (OR 11.6, *p* = 0.0008) and aminoglycosides (OR 2.14, *p* = 0.0001) than in community patients. Over time, there has been a significant increase in resistance to penicillin/inhibitor associations in *K. pneumoniae* isolated from nosocomial samples (*p* = 0.0137, *p* = 0.0075, *p* < 0.0001) compared to community isolates (Table 3 panel B). A notable increase in resistance to aminoglycosides was observed among Gram-negative K. pneumoniae isolates from samples obtained from nosocomial patients when compared with data obtained from community isolates in 2019 (OR = 2.34, *p* = 0.0170). This difference exhibited fluctuations, demonstrating a tendency to disappear in 2020 before re-emerging in 2021 (OR 3.5—*p* value 0.0122), where it subsequently returned to being non-significant in 2022. In 2019, noteworthy resistance to imipenem (OR 8.2—*p* value 0.0063) and trimethoprim/sulfamethoxazole (OR 3.14—*p* value 0.0124) was observed in nosocomial samples when compared to community samples. Additionally, notable resistance to penicillin/inhibitors was observed among nosocomial isolates in the years 2020 (OR 3—*p* value 0.0137), 2021 (OR 2.4—*p* value 0.0075) and 2022 (OR 4—*p* value < 0.0001), indicating an inclination of this phenomenon to increase over time. Finally, an elevated level of resistance to ciprofloxacin was observed among hospitalized patients compared to data recorded in the community, specifically for the year 2021 (Table 3 panel C). Statistically significant differences are indicated in bold. The isolates were categorized according to their antibiotic resistance profiles, with the results grouped into two distinct clusters: multidrug-resistant (MDR) and non-multidrug-resistant (non-MDR) (Figure 2). Subsequently, the groups underwent further analysis to ascertain their capacity for biofilm production. A total of 140 isolates were selected for analysis, of which 113 were identified as gram-negative bacteria. Subsequently, the isolates were categorized as follows: a total of 50 *K. pneumoniae* isolates were characterized, of which 37 were identified as originating from nosocomial sources and 13 from community samples. Similarly, 48 *E. coli* isolates were identified, of which 34 were of nosocomial origin and 14 were of community origin. In addition, ten isolates of *P. aeruginosa* were identified, of which nine were sourced from nosocomial settings and one from the community. Lastly, five isolates of *Proteus* spp. were identified, all of which originated from nosocomial sources. Of the total of 140 isolates, 27 were identified as gram-positive and were categorized as follows: a total of 15 *Enterococcus* spp. were characterized, of which 11 were identified as originating from nosocomial sources and 4 were from community samples. In addition, 12 isolates of *Staphylococcus* spp. were identified, of which 8 were sourced from nosocomial settings and 4 were from the community.

**Figure 3:**
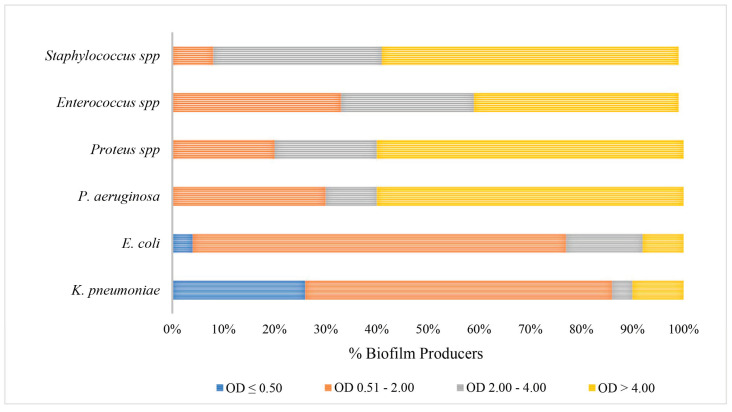
Comparison of biofilm production rates in different gram-positive and gram-negative bacterial species.

**Figure 4:**
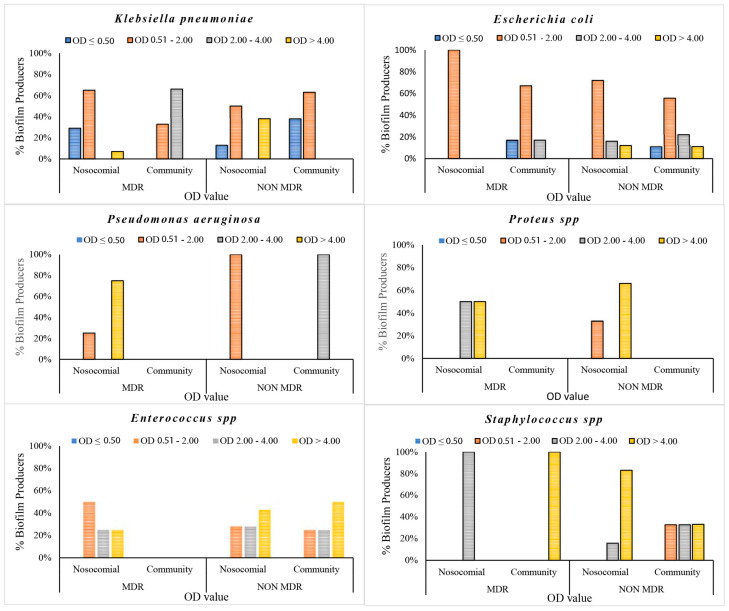
Biofilm production percentages of gram-negative and gram-positive bacteria isolated in both community and nosocomial settings.

The data demonstrated a significant disparity in biofilm formation capabilities among the strains, which were classified as strong, medium and low. It is noteworthy that there was an inverse relationship between the prevalence of strong biofilm-producing strains and antibiotic resistance among the species *K. pneumoniae*, *Enterococcus* spp. and *Staphylococcus* spp. (Figure 2). Medium biofilm-producing *E. coli* strains (OD = 2.00–4.00), which were sensitive to the majority of the tested antibiotics, exhibited a higher formation rate than strains resistant to cephalosporins, fluoroquinolones and trimethoprim/sulfamethoxazole. In addition, our data indicate that *K. pneumoniae* strains exhibited low biofilm production in 62% and 56% of MDR and NON-MDR isolates, respectively. The proportion of strong NON-MDR-producing strains was higher (19%) than that of MDR strains (6%) (Figure 2). The gram-positive Enterococcus spp. exhibited a noteworthy capacity for biofilm formation, with 25% and 46% strong production and 25% and 27% medium production of the MDR and NON-MDR strains, respectively. Furthermore, a strong capacity for biofilm production was observed in 33% and 66% of the MDR and NON-MDR isolated strains of *Staphylococcus* spp., respectively (Figure 2). Among the isolates studied, the strains of *Proteus* spp. and *P. aeruginosa* proved to be the major producers of biofilm, followed closely by the Gram-positive ones. Specifically, *P. aeruginosa* and *Proteus* spp. showed 60% strong producing isolates, followed by gram-positives, among which *Staphylococcus* spp. stood out, with strong medium production rates of 58% and 33%. Only 10% of *K. pneumoniae* and 8% of *E. coli* strains showed strong biofilm production (Figure 3). Isolates from community patients showed a low capacity for biofilm production compared to microorganisms isolated from hospital patients, where we observed a greater prevalence of medium and strong biofilm production (Figure 4). The data show that *K. pneumoniae* had a higher percentage of medium production (100%) in MDR strains of community origin. NON MDR *E. coli* isolates showed similar biofilm formation capacity in both nosocomial (medium 16%–strong 12%) and community settings (medium 22%–strong 11%), while medium major production capacity was detected in MDR strains at the community level (17%). Among the NON MDR strains of *P. aeruginosa*, a high percentage of biofilm formation was highlighted in the medium range in the community setting (100%). Among gram-positives, 25% and 43% *Enterococcus* spp. isolated at the nosocomial level, MRD and NON MDR, respectively, showed strong biofilm production. These data highlight how strains that show lower resistance have a greater capacity for biofilm formation. The percentages of isolated strains which were strong producers of NON MDR biofilms, such as *Staphylococcus* spp., were more prevalent at the nosocomial level than at the community level (83% and 33%, respectively) (Figure 4).

## 4. Discussion

UTI is a common infectious disease that requires medical treatment in both outpatient and inpatient settings. It represents an economic burden of more than USD 2 billion annually in the United States and several billion dollars worldwide [28]. Based on current guidelines, mild UTIs may resolve without antibiotic treatment. It is important to avoid misuse of antibiotics in such cases, as this can lead to the emergence of resistant strains of uropathogens, making them more challenging to treat. Despite improvements in the pharmaceutical management of UTI patients in routine clinical practice, UTI pathogen isolation rates have not decreased, nor has the spread of antimicrobial resistance (AMR) in UTIs been contained [34]. This may be partly due to improved care resulting in a steady increase in the number of elderly patients with recurrent UTIs. Consistent with previous research, our data indicates that gram-negative bacteria from hospital-acquired UTIs were more resistant to aminoglycosides (OR 2.24), ciprofloxacin (OR 1.5), ESBLs (OR 1.8), phosphomycin (OR 1.8), trimethoprim/sulfamethoxazole (OR 1.6) and imipenem (OR 13.4) than community isolates. Among the gram-negative strains tested, *K. pneumoniae* isolated from nosocomial samples was significantly more resistant to penicillin/inhibitor combinations, trimethoprim-sulfamethoxazole and ciprofloxacin than that isolated from community patients. Our data are similar to the results of other studies that have been conducted in Saudi Arabia, China and Italy [17,28]. The study findings indicate that imipenem and aminoglycosides were the most effective antimicrobial agents against the main uropathogens isolated in both community and hospital settings. These alternatives can be used instead of β-lactams and fluoroquinolones when resistance to first-line UTI agents is endemic. According to our data, after *E. coli* and *K. pneumoniae*, *P. aeruginosa* is the third most commonly isolated organism in hospitalized patients with UTIs. We found a higher prevalence of *P. aeruginosa* in hospital settings compared to the community [30]. This microorganism is a common cause of biofilm-mediated infections, including catheter-associated urinary tract infections, and is often a cause of hospital-acquired infections [30]. *E. faecalis* was the most commonly isolated gram-positive bacterium, followed by *E. faecium*. Moreover, *E. faecium* exhibited high resistance to ampicillin/sulbactam and imipenem. The capacity for biofilm production was observed to be significantly lower in isolates from community patients when compared to those isolated from nosocomial patients, where a higher prevalence of medium and strong biofilm production was noted. These findings support the hypothesis that biofilm production is a crucial element in the pathogenesis of bacterial nosocomial disease. The data analysis demonstrated that *Proteus* spp. and *P. aeruginosa* strains were the predominant strong biofilm producers, followed by gram-positive strains such as *Staphylococcus* spp., which exhibited high and medium biofilm production rates of 58% and 33%, respectively. In contrast, only 10% of *K. pneumoniae* strains and 8% of *E. coli* demonstrated evidence of strong biofilm production. *E. coli* strains that demonstrated susceptibility to the majority of the antibiotics under investigation exhibited an enhanced capacity for biofilm formation in comparison to the strains that exhibited resistance to cephalosporins, fluoroquinolones and trimethoprim/sulphamethoxazole. Additionally, an inverse correlation was observed between antibiotic sensitivity and biofilm formation for *K. pneumoniae*. This NON-MDR strains that were sensitive to the majority of antibiotics tested demonstrated high biofilm production. The data presented here are consistent with the findings of other studies conducted in Egypt on *E. coli* uropathogens [35]. In contrast, to the best of our knowledge, this phenomenon has not previously been described in our country. This evident correlation between biofilm formation and resistance suggests that there may be a cost associated with resistance at the cellular level, and that isolates with lower resistance may rely on biofilms to enhance their survival. The inverse correlation between antibiotic resistance and biofilm formation underscores the necessity of evaluating biofilm formation capabilities in clinical settings rather than relying exclusively on antibiotic susceptibility testing. This approach may offer insights into the challenges associated with antibiotic therapy and the persistence of infections despite in vitro susceptibility. The management of UTIs has become a significant challenge in light of the ongoing dissemination of AMR uropathogens. It is imperative that we intensify our efforts aimed at limiting the spread of AMR bacteria. This entails the prudent and selective usage of antibiotics and the implementation of effective infection control procedures, as well as the surveillance of local UTI etiologies.

## 5. Conclusions

Our findings demonstrate that gram-negative bacteria exhibit greater overall antimicrobial resistance than gram-positive bacteria in both hospital and community isolates. Additionally, isolates from community samples exhibited limited capacity for biofilm production, in contrast to those from hospital samples, where medium and strong biofilm formations were more prevalent. Our data also highlight the inverse correlation between resistance and capacity for biofilm formation, with strains displaying lower levels of resistance having greater ability to form biofilms. In light of the considerable challenge posed by antimicrobial resistance in hospital and care settings, there is a pressing need to refine the management and control of UTI through antibiotic therapy that takes into account local epidemiological trends.

## Figures and Tables

**Table 1:** The most common pathogens isolated in the hospital (upper panel) and community (lower panel) setting over a four-year period.

Hospital	2019	2020	2021	2022	2023
*E. coli*	220	121	160	138	173
*K. pneumoniae*	115	87	125	112	184
*P. aeruginosa*	44	22	35	32	39
*E. faecium*	21	19	13	7	4
*E. faecalis*	31	20	28	17	25
**Community**	**2019**	**2020**	**2021**	**2022**	**2023**
*E. coli*	140	77	94	107	149
*K. pneumoniae*	40	35	37	49	73
*P. aeruginosa*	11	5	9	5	9
*E. faecium*	2	0	0	0	0
*E. faecalis*	12	6	5	11	8

**Table 2:** (**A**). Resistance rates in nosocomial isolates (%). (**B**). Resistance rates in community isolates (%).

(A)				
NOSOCOMIAL	*E. coli*	*K. pneumoniae*	*P. aeruginosa*	*A. baumannii Complex*
PENICILLIN/INHIBITOR	31%	66.4%		
CIPROFLOXACIN	41%	59%	25%	98%
ESBL	44%	78%		
PHOSFOMYCIN	3.30%	42%	9.30%	
AMINOGLICOSIDES	10%	27.40%	20%	87%
IMIPENEM	3%	33%	14%	100%
TRIMETHOPRIM/Sulfamethoxazole	29%	43%		87%
PIPERICILLIN/TAZOBACTAM			16%	
TOBRAMICIN				100%
**(B)**				
**COMMUNITY**	***E. coli***	***K. pneumoniae***	***P. aeruginosa***	***A. baumannii Complex***
PENICILLIN/INHIBITOR	29%	44%		
CIPROFLOXACIN	35%	56%	26%	50%
ESBL	32%	52%		
PHOSFOMYCIN	5%	16%	40%	
AMINOGLYCOSIDES	6.20%	47%	7.3%	0
IMIPENEM	0	4%	0	100%
TRIMETHOPRIM/Sulfamethoxazole	28%	30%		0
PIPERICILLIN/TAZOBACTAM			24%	
TOBRAMICIN				0

**Table 3:** (**A**). Percentage of resistance in gram-negative isolates from community and hospital settings. (**B**). Odds ratios for the rates of resistance in isolates detected in the community and in hospital-acquired isolates. Statistically significant differences are indicated in bold. (**C**). Odds ratios for the rate of resistance in *K. pneumoniae* isolates detected in the community and in hospital-acquired isolates. Statistically significant differences are indicated in bold.

(A)					
Gram-Negative Resistance					
	Nosocomial		Community		
aminoglycosides	19%		7.3%		
penicillin/inhibitor	45%		31%		
phosfomycin	28%		13%		
imipenem	19%		23%		
trimethoprim/Sulfamethoxazole	36%		29%		
ciprofloxacin	44%		42%		
(**B**)					
	***E. coli***	***K. pneumoniae***		***P. aeruginosa***	
Aminoglycosydes	OR 1.141 *p* = 0.503	OR 2.14 ***p* = 0.0001**		OR 1.58 *p* = 0.406	
Penicillin/Inhibitor	OR 1.04 *p* = 0.692	OR 2.40 ***p* ˂ 0.0001**		OR 0.48 *p* = 0.256	
Phosfomycin	OR 1.211 *p* = 0.694	OR 1.03 *p* = 0.916		OR 2.5 *p* = 0.183	
Imipenem	OR 1.302 *p* = 0.8717	OR 11.6 ***p* = 0.0008**		OR 0.37 *p* = 0.463	
Trimethoprim/ Sulfamethoxazole	OR 1.142 *p* = 0.395	OR 1.84 ***p* = 0.0036**		NA	
Ciprofloxacin	OR 1.07 *p* = 0.629	OR 1.93 ***p* = 0.0008**		OR 0.537 *p* = 0.174	
(**C**)					
	***Klebsiella pneumoniae***				
	**2019**	**2020**	**2021**		**2022**
Aminoglycosides	**OR 2.34** (CI 95% 1.1646–4.721) ***p* = 0.0170**	OR 2 (IC 95% 0.8341–4.470) *p* = 0.1244	**OR 3.5** (IC 95% 1.3105–9.161) ***p* = 0.0122**		OR 2 (IC 95% 0.8388–3.688) *p* = 0.1350
Penicillin/Inhibitor	OR 1.7 (IC 95% 0.9691–2.8937) *p* = 0.0647	**OR 3** (IC 95% 1.236–6.450) ***p* = 0.0137**	**OR 2.4** (IC 95% 1.2580–4.450) ***p* = 0.0075**		**OR 4** (IC 95% 1.9624–6.923) ***p* ˂ 0.0001**
Ciprofloxacin	OR 1.8 (IC 95% 0.8509–3.9918) *p* = 0.1210	OR 3 (IC 95% 0.9426–6.851) *p* = 0.0653	**OR 3** (IC 95%1.2751–6.178) ***p* = 0.0104**		OR 1.52 (IC 95% 0.7490–3.095) *p* = 0.2454
ESBL	**OR 2.6** (IC 95% 1.1807–5.6576) ***p* = 0.0175**	**OR 3** (IC 95% 1.2061–7.422) ***p* = 0.0181**	**OR 2.2** (IC 95% 1.1250–4.325) ***p* = 0.0213**		**OR 3.1** (IC 95% 1.6288–6.0663) ***p* = 0.0006**
Phosfomycin	OR 0.9 (IC 95% 0.4109–1.94434) *p* = 0.7766	OR 0.24 (IC 95% 0.0340–1.727) *p* = 0.1572	OR 1.8 (IC 95% 0.5118–6.869) *p* = 0.3427		OR 1.1 (IC 95% 0.2451–4.573) *p* = 0.9390
Imipenem	**OR 8.2** (IC 95% 1.8226–36.726) ***p* = 0.0063**	OR 6 (IC 95% 0.2815–113.337) *p* = 0.2578	OR 8 (IC 95% 0.4417–140.495) *p* = 0.1603		OR 6 (IC 95% 0.3155–106.331) *p* = 0.2368
Trimethoprim/ Sulfamethoxazole	**OR 3.14** (IC 95% 1.2805–7.697) ***p* = 0.0124**	OR 2.3 (IC 95% 0.8538–6.3436) *p* = 0.0987	OR 1.3 (IC 95% 0.5623–2.8937) *p* = 0.5302		OR 2 (IC 95% 0.8840–3.7561) *p* = 0.1040

## Data Availability

No new data were created or analyzed in this study. Data sharing is not applicable to this article.

## References

[1] Mancuso G., Trinchera M., Midiri A., Zummo S., Vitale G., Biondo C. (2024). Novel Antimicrobial Approaches to Combat Bacterial Biofilms Associated with Urinary Tract Infections. Antibiotics.

[2] Truong W.R., Yamaki J. (2019). Re: Recurrent Uncomplicated Urinary Tract Infections in Women: AUA/CUA/SUFU Guideline. J. Urol..

[3] Glover M., Moreira C.G., Sperandio V., Zimmern P. (2014). Recurrent urinary tract infections in healthy and nonpregnant women. Urol. Sci..

[4] Bergamin P.A., Kiosoglous A.J. (2017). Surgical management of recurrent urinary tract infections: A review. Transl. Androl. Urol..

[5] Mancuso G., Midiri A., Gerace E., Marra M., Zummo S., Biondo C. (2023). Urinary Tract Infections: The Current Scenario and Future Prospects. Pathogens.

[6] Flores-Mireles A.L., Walker J.N., Caparon M., Hultgren S.J. (2015). Urinary tract infections: Epidemiology, mechanisms of infection and treatment options. Nat. Rev. Microbiol..

[7] Werneburg G.T. (2022). Catheter-Associated Urinary Tract Infections: Current Challenges and Future Prospects. Res. Rep. Urol..

[8] Govindarajan D.K., Kandaswamy K. (2022). Virulence factors of uropathogens and their role in host pathogen interactions. Cell Surf..

[9] Zhou Y., Zhou Z., Zheng L., Gong Z., Li Y., Jin Y., Huang Y., Chi M. (2023). Urinary Tract Infections Caused by Uropathogenic Escherichia coli: Mechanisms of Infection and Treatment Options. Int. J. Mol. Sci..

[10] Tiseo G., Brigante G., Giacobbe D.R., Maraolo A.E., Gona F., Falcone M., Giannella M., Grossi P., Pea F., Rossolini G.M. (2022). Diagnosis and management of infections caused by multidrug-resistant bacteria: Guideline endorsed by the Italian Society of Infection and Tropical Diseases (SIMIT), the Italian Society of Anti-Infective Therapy (SITA), the Italian Group for Antimicrobial Stewardship (GISA), the Italian Association of Clinical Microbiologists (AMCLI) and the Italian Society of Microbiology (SIM). Int. J. Antimicrob. Agents.

[11] van Belkum A., Bachmann T.T., Ludke G., Lisby J.G., Kahlmeter G., Mohess A., Becker K., Hays J.P., Woodford N., Mitsakakis K. (2019). Developmental roadmap for antimicrobial susceptibility testing systems. Nat. Rev. Microbiol..

[12] Sachdev C., Anjankar A., Agrawal J. (2022). Self-Medication With Antibiotics: An Element Increasing Resistance. Cureus.

[13] Almagor J., Temkin E., Benenson I., Fallach N., Carmeli Y., The DRIVE-AB Consortium (2018). The impact of antibiotic use on transmission of resistant bacteria in hospitals: Insights from an agent-based model. PLoS ONE.

[14] Biondo C. (2023). New Insights into the Pathogenesis and Treatment of Urinary Tract Infections. Pathogens.

[15] Tang K.W.K., Millar B.C., Moore J.E. (2023). Antimicrobial Resistance (AMR). Br. J. Biomed. Sci..

[16] Barchitta M., Quattrocchi A., Maugeri A., La Rosa M.C., La Mastra C., Sessa L., Cananzi P., Murolo G., Oteri A., Basile G. (2019). Antibiotic Consumption and Resistance during a 3-Year Period in Sicily, Southern Italy. Int. J. Environ. Res. Public Health.

[17] Maione A., Galdiero E., Cirillo L., Gambino E., Gallo M.A., Sasso F.P., Petrillo A., Guida M., Galdiero M. (2023). Prevalence, Resistance Patterns and Biofilm Production Ability of Bacterial Uropathogens from Cases of Community-Acquired Urinary Tract Infections in South Italy. Pathogens.

[18] Gaurav A., Bakht P., Saini M., Pandey S., Pathania R. (2023). Role of bacterial efflux pumps in antibiotic resistance, virulence, and strategies to discover novel efflux pump inhibitors. Microbiology.

[19] Maharjan G., Khadka P., Siddhi Shilpakar G., Chapagain G., Dhungana G.R. (2018). Catheter-Associated Urinary Tract Infection and Obstinate Biofilm Producers. Can. J. Infect. Dis. Med. Microbiol..

[20] Uruen C., Chopo-Escuin G., Tommassen J., Mainar-Jaime R.C., Arenas J. (2020). Biofilms as Promoters of Bacterial Antibiotic Resistance and Tolerance. Antibiotics.

[21] Sharma S., Mohler J., Mahajan S.D., Schwartz S.A., Bruggemann L., Aalinkeel R. (2023). Microbial Biofilm: A Review on Formation, Infection, Antibiotic Resistance, Control Measures, and Innovative Treatment. Microorganisms.

[22] Davies J., Davies D. (2010). Origins and evolution of antibiotic resistance. Microbiol. Mol. Biol. Rev..

[23] Danese P.N. (2002). Antibiofilm approaches: Prevention of catheter colonization. Chem. Biol..

[24] Kouchak F., Askarian M. (2012). Nosocomial infections: The definition criteria. Iran. J. Med. Sci..

[25] Pirkani G.S., Awan M.A., Abbas F., Din M. (2020). Culture and PCR based detection of bacteria causing urinary tract infection in urine specimen. Pak. J. Med. Sci..

[26] Mancuso G., Midiri A., Zummo S., Gerace E., Scappatura G., Biondo C. (2021). Extended-spectrum beta-lactamase & carbapenemase-producing fermentative Gram-negative bacilli in clinical isolates from a University Hospital in Southern Italy. New Microbiol..

[27] Gaur P., Hada V., Rath R.S., Mohanty A., Singh P., Rukadikar A. (2023). Interpretation of Antimicrobial Susceptibility Testing Using European Committee on Antimicrobial Susceptibility Testing (EUCAST) and Clinical and Laboratory Standards Institute (CLSI) Breakpoints: Analysis of Agreement. Cureus.

[28] Alamri A., Hamid M.E., Abid M., Alwahhabi A.M., Alqahtani K.M., Alqarni M.S., Abomughaid M. (2018). Trend analysis of bacterial uropathogens and their susceptibility pattern: A 4-year (2013–2016) study from Aseer region, Saudi Arabia. Urol. Ann..

[29] Serretiello E., Folliero V., Santella B., Giordano G., Santoro E., De Caro F., Pagliano P., Ferro M., Aliberti S.M., Capunzo M. (2021). Trend of Bacterial Uropathogens and Their Susceptibility Pattern: Study of Single Academic High-Volume Center in Italy (2015–2019). Int. J. Microbiol..

[30] Kasew D., Desalegn B., Aynalem M., Tila S., Diriba D., Afework B., Getie M., Biset S., Baynes H.W. (2022). Antimicrobial resistance trend of bacterial uropathogens at the university of Gondar comprehensive specialized hospital, northwest Ethiopia: A 10 years retrospective study. PLoS ONE.

[31] Stepanovic S., Vukovic D., Hola V., Di Bonaventura G., Djukic S., Cirkovic I., Ruzicka F. (2007). Quantification of biofilm in microtiter plates: Overview of testing conditions and practical recommendations for assessment of biofilm production by staphylococci. J. Pathol. Microbiol. Immunol..

[32] Christensen G.D., Baldassarri L., Simpson W.A. (1995). Methods for studying microbial colonization of plastics. Methods Enzymol..

[33] Szumilas M. (2010). Explaining odds ratios. J. Can. Acad. Child Adolesc. Psychiatry.

[34] Chowdhury S.S., Tahsin P., Xu Y., Mosaddek A.S.M., Muhamadali H., Goodacre R. (2024). Trends in Antimicrobial Resistance of Uropathogens Isolated from Urinary Tract Infections in a Tertiary Care Hospital in Dhaka, Bangladesh. Antibiotics.

[35] Alshaikh S.A., El-Banna T., Sonbol F., Farghali M.H. (2024). Correlation between antimicrobial resistance, biofilm formation, and virulence determinants in uropathogenic Escherichia coli from Egyptian hospital. Ann. Clin. Microbiol. Antimicrob..

